# Genome-wide identification and functional prediction of nitrogen-responsive intergenic and intronic long non-coding RNAs in maize (*Zea mays* L.)

**DOI:** 10.1186/s12864-016-2650-1

**Published:** 2016-05-11

**Authors:** Yuanda Lv, Zhikai Liang, Min Ge, Weicong Qi, Tifu Zhang, Feng Lin, Zhaohua Peng, Han Zhao

**Affiliations:** Provincial Key Laboratory of Agrobiology, Institute of Biotechnology, Jiangsu Academy of Agricultural Sciences, Nanjing, China; Department of Biochemistry and Molecular Biology, Mississippi State University, Mississippi, Mississippi State USA

**Keywords:** RNA-Seq, Long noncoding RNA, Nitrogen response, Coexpression network

## Abstract

**Background:**

Nitrogen (N) is an essential and often limiting nutrient to plant growth and development. Previous studies have shown that the mRNA expressions of numerous genes are regulated by nitrogen supplies; however, little is known about the expressed non-coding elements, for example long non-coding RNAs (lncRNAs) that control the response of maize (*Zea mays* L.) to nitrogen. LncRNAs are a class of non-coding RNAs larger than 200 bp, which have emerged as key regulators in gene expression.

**Results:**

In this study, we surveyed the intergenic/intronic lncRNAs in maize B73 leaves at the V7 stage under conditions of N-deficiency and N-sufficiency using ribosomal RNA depletion and ultra-deep total RNA sequencing approaches. By integration with mRNA expression profiles and physiological evaluations, 7245 lncRNAs and 637 nitrogen-responsive lncRNAs were identified that exhibited unique expression patterns. Co-expression network analysis showed that the nitrogen-responsive lncRNAs were enriched mainly in one of the three co-expressed modules. The genes in the enriched module are mainly involved in NADH dehydrogenase activity, oxidative phosphorylation and the nitrogen compounds metabolic process.

**Conclusions:**

We identified a large number of lncRNAs in maize and illustrated their potential regulatory roles in response to N stress. The results lay the foundation for further in-depth understanding of the molecular mechanisms of lncRNAs’ role in response to nitrogen stresses.

**Electronic supplementary material:**

The online version of this article (doi:10.1186/s12864-016-2650-1) contains supplementary material, which is available to authorized users.

## Background

Maize (*Zea mays* L.) is an important cereal grain crop that is grown widely in various agro-ecological environments worldwide. Apart from being used as human food, maize is also largely used for livestock feed and industrial materials in developed countries [1]. Plant growth and grain development require an abundant supply of nutrients particularly nitrogen (N) [2–4]. As a major yield-determining factor, N is a vital plant nutrient for plant growth and development. It not only provides an N source for amino acids, nucleic acids, chlorophyll and ATP (adenosine triphosphate) [5–7], but also mediates the utilization of phosphorus, potassium and other nutrients in plants [8]. Massive amounts of synthetic nitrogen fertilizer are currently applied to crop fields, however, the crops do not use significant amounts. The excess nitrogen inevitably becomes a major component of global environmental pollution [9–12]. To reduce nitrogen pollution while increasing productivity, enabling crops to use nitrogen more efficiently is critical [13–15]. To achieve this goal, multiple quantitative trait loci (QTLs) in the related pathways have been identified [16–19] and differential gene expression studies in response to nitrogen resources and stresses have been reported [20–24]. However, nitrogen assimilation and its associated metabolic pathways are highly complicated. The underlying molecular mechanism of nitrogen regulation remains largely unknown [25–27].

In recent years, studies of long non-coding RNAs (lncRNAs), a class of non-coding RNA molecules longer than 200 nt, have extended our understanding of the eukaryotic transcriptome. Studies in animals and humans have indicated that lncRNAs play a key role in regulating diverse biological processes, such as transcriptional regulation, dosage compensation and genomic imprinting [28–31]. However, the study of lncRNAs in plants remains largely unexplored. With the rapid development of high-throughput sequencing technologies, large numbers of lncRNAs have been identified *in silico* or experimentally in *Arabidopsis*, maize, rice and other plant species. Boerner and McGinnis [32] identified 1802 potential lncRNAs in the maize genome using a computational pipeline from 18,668 full-length cDNA sequences. Li [33] identified 20,163 putative lncRNAs and 1704 high-confidence lncRNAs by exploiting available public expressed sequence tag (EST) databases and RNA-seq datasets from 30 different experiments. In addition, several studies have demonstrated the regulation of lncRNA expression in response to abiotic stresses in plants [34–38]. Through *in silico* genome-wide analysis of *Arabidopsis* full-length cDNA databases, 76 ncRNAs including five siRNA precursors and 14 natural antisense transcripts of protein-coding genes were identified. Twenty-two lncRNAs related to abiotic stress response were identified by studying stress expression profiles [39]. Zhang et al. [40] reported the deep sequencing of mRNAs derived from drought-stressed maize and 1724 lncRNAs were identified as drought-responsive. In eukaryotes, most transcripts, including mRNAs and lncRNAs have a polyadenylated (poly (A)+) tail at their 3′ ends. However, non-polyadenylated transcripts, such as ribosomal RNAs (rRNAs) and replication-dependent histone mRNAs end in a conserved 3′ stem-loop sequence [41], and some lncRNAs [42–44] transcribed by RNA polymerase II are present in large numbers among transcripts. Yang et al. [42] explored poly(A) + and poly(A)- RNAs from HeLa cells and H9 human embryonic stem cells using deep sequencing, and found that a few excised introns accumulate in cells and constitute a new class of non-polyadenylated lncRNAs. Di et al. [44] performed total RNA-seq for seedlings of *Arabidopsis thaliana* under four stress conditions and identified 245 poly (A) + and 58 poly (A)- lncRNAs that are differentially expressed under various stress stimuli. Thus, total RNA sequencing should help us to detect more lncRNAs because it selects RNAs independent of the presence of a poly(A) tail.

To investigate the potential role of lncRNAs with/without polyA tails in response to nitrogen resources, we first performed ultra-deep total RNA sequencing with only rRNA depletion and identified a collection of intergenic/intronic lncRNAs genes expressed in maize leaves. Among the 7245 identified putative lncRNAs, 637 were nitrogen-responsive. Eighty-five differentially expressed N responsive lncRNAs were further classified into three co-expressed modules, some of which are involved in metabolic processes associated with energy, oxidative phosphorylation, phosphorus and nitrogen compounds. These results suggest that lncRNAs might have unique roles in the response to nitrogen.

## Methods

### Plant materials and nitrogen stress treatments

The elite maize inbred line B73 was germinated in a plastic container (20 × 20 × 40 cm) filled with a mixture of quartz sand (80 %) and vermiculite (20 %) in a greenhouse at JAAS (Jiangsu Academy of Agricultural Sciences). Plants were watered daily until the three-leaf stage and then watered with nutrient solution containing 5 mM CaCl_2_, 0.5 mM KH_2_PO_4_, 2 mM MgSO_4_, 0.05 mM EDTA-Fe-Na Salt, 10 uM MnCl_2_, 0.3 uM CuSO_4_, 1 uM ZnSO_4_, 50 uM H_3_BO_3_ and 0.5 uM Na_2_MoO_4_. Two different nitrate (KNO_3_) concentrations were used: one as sufficient N conditions (15 mM) and one as limited N conditions (0.15 mM). Leaves at the seven-leaf (V7) stage were collected and stored at −80 °C for further analysis. Three independent biological replicates were grown to validate RNA expression via quantitative real-time PCR (qPCR).

### Leaf N content measurement

To analyze the total nitrogen content of the leaves, B73 leaves from the two nitrogen treatments were dried at 105 °C until their weight was stable. They were then ground to fine powder to ensure digestion. Total N contents were then determined using San^++^ Continuous Flow Analyzer (Skalar, the Netherlands).

### RNA extraction, rRNA depletion and total RNA-seq sequencing

The total RNA of each sample was isolated using a Takara MiniBEST universal RNA extraction kit (Takara, Japan) and digested with RNase-free DNase (Qiagen, Germany) according to the manufacturer’s protocol. RNA was then purified and concentrated using an RNeasy column (Takara). The RNA concentration and quality were evaluated using an Agilent 2100 Bioanalyzer (Agilent Technologies, USA). RNA samples were treated with the RiboMinus™ plant rRNA removal kit (Invitrogen, CA, USA) for rRNA depletion. Total RNA-seq libraries were then constructed and sequenced using an Illumina HiSeq™ 2500 with paired-end method by Berry Genomics Co. Ltd., China.

### RNA-seq analysis

The Tuxedo suite [45] was used to complete RNA-seq analysis in this study. First, the FastQC [46] program was employed to assess the quality of the reads. The SolexaQA++ v3.1 [47] program was introduced to perform quality trimming using the Q20 value (Phred score 20, 1 in 100 chance of incorrect base call). Any reads less than 40 bp were removed after trimming. Cleaned reads were then aligned to the ribosomal RNA (rRNA) sequence databases using the BWA [48] program with default parameters. Any reads containing rRNA sequences were removed and the remaining reads were used for further analysis. The maize genome assembly B73 (v3) was downloaded from the Plant Ensembl database (http://plants.ensembl.org, v26), which contains 39,556 genes and 63,174 transcripts. Cleaned reads were aligned to the maize reference genome using Tophat v2.0.13 [49]. Low-quality alignment results were filtered out using Samtools [50] with a mapping quality threshold of 20. After the alignment, Cufflinks (version 2.1.1) [45] was used to assemble reads into transcripts. Subsequently, the assembled transcripts were merged using Cuffmerge [45] to obtain a non-redundant unified set of transcripts. Transcripts from the two assemblies were compared with reference annotation using the Cuffcompare [45] program. The class codes in the Cuffcompare output were used to generate a consensus assembly. The number of fragments per kilobase per million mapped reads (FPKM) per gene were calculated using cuffquant [45]. Cuffdiff [45] was used to perform pairwise comparisons between samples to identify differentially expressed transcripts. Cuffdiff was used to test for differential expression and regulation among the assembled transcripts across the different samples using the Cufflinks output.

### Computational identification of intergenic and intronic lncRNAs

To find lncRNAs, a strict computational strategy was performed as described by Iyer et al. [51] and Xiao et al. [52]. First, transcripts with class code “I” and “U” were selected for further non-coding analysis. The Gffread program was used to extract these transcripts. Then, these transcripts were aligned against uniref90 and Pfam protein databases using the CPC [53] program to assess their protein-coding potential. Non-coding transcripts larger than 200 bp, with an FPKM > 1 and a CPC score < -1, were finally considered as candidate lncRNAs for further analysis.

### Co-expression network analysis

To construct the co-expression network, a set of microarray data under limited and sufficient nitrogen conditions in maize produced by a previous study [54] were employed. The data set generated 90 gene expression profiles containing 84,246 probe sets using the Affymetrix chip platform. Combined expression profiles were extracted and normalized using the GEOquery [55] package. Co-expression correlation between lncRNA probes and non-lncRNA probes was then calculated using Pearson correlation with R^2^ ≥ 0.85. The normalized expression data from lncRNAs probes and co-expressed probes were extracted to construct an unsigned co-expression network using the WGCNA [56] package with a soft threshold = 8. Module assignment of lncRNAs and non-lncRNAs was identified using Topological Overlap Matrix (TOM). Eigengenes of each module were evaluated and the co-expressed network was visualized by the Cytoscape [57] program.

### GO (Gene Ontology) term enrichment analysis

The eigengene probes of each module were assigned putative functions by searching using the Blastx [58] program against the uniprot protein database [59], using a cut-off e-value ≤ 1e^-15^. Coding eigengenes were then submitted to AgriGO online toolkits [60] for gene ontology term enrichment. Fisher’s exact test was applied for the enrichment analysis and the false discovery rate (FDR) was assessed using the Bonferroni method. The significance level was set to 0.1 to identify the significant functional terms.

### Validation and quantification of lncRNAs

To validate the lncRNAs, 14 lncRNAs were selected and subjected to a PCR test using B73 genomic DNA and cDNA to validate the accuracy of the assembly. Primers were designed using mInDel [61] are shown in Additional file 1: Table S1 and Additional file 2: Table S2. To confirm the RNA-seq expression results, total RNA was used to synthesize cDNA using a PrimeScript™ RT reagent kit (Takara) and random hexamer primers. Ubiquitin was used as the internal reference gene control [62]. qPCR was performed using SYBR Premix DimerEraser™ kits (Takara) on a Real Time PCR System (Roche LightCycler^R^ 96, USA), according to the manufacturer’s instructions. Quantification results of target transcripts were calculated using the comparative ΔΔCt method. Three biological replicates for each selected transcript were used.

## Results

### Phenotypic and physiological responses of maize seedlings to N stress

**Fig. 1:**
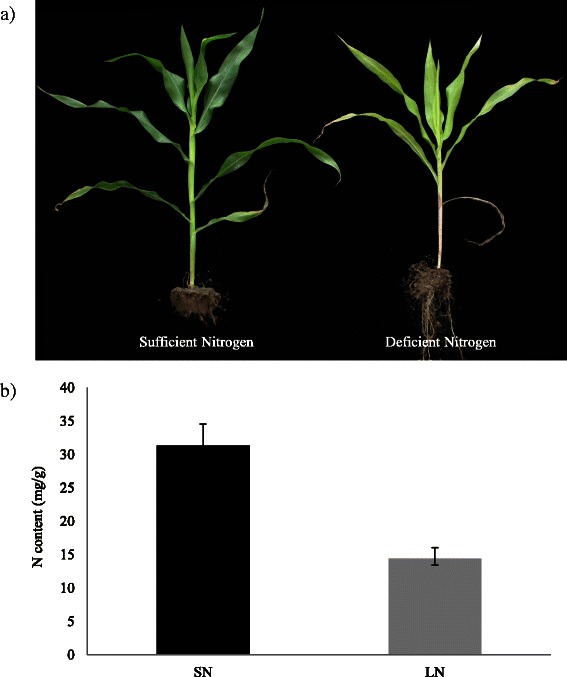
Phenotypic and physiological changes in response to N stress. **a** Phenotypes in response to N stress; **b**) Leaf N content at seven-leaf (V7) stage under two N conditions. Bars represent the standard error of three biological replicates

The influence of N stress of varied intensities on developing maize seedlings (inbred line B73) was monitored by measuring the N content in the leaves. Before N stress, there was no significant difference detected between sufficient nitrogen and limited nitrogen treatments. After treatment, the leaf color differed between sufficient nitrogen and limited nitrogen supplies (Fig. 1a). Measurement of the leaf N content demonstrated that the N content in the nitrogen-limited maize leaves was significantly lower than that in the nitrogen-sufficient treatment. The results suggested that seedlings of inbred line B73 were sensitive to the N stress and that the internal N content was altered by the N treatments (Fig. 1b).

### Ultra-deep total RNA sequencing and mapping

To obtain a comprehensive understanding of the transcriptome under nitrogen stress, total RNAs of V7 leaves were isolated from the maize inbred line B73 grown under sufficient and limited N conditions. RNA-Seq libraries were constructed from the total RNA with rRNA depletion and sequenced by the paired-end method (100 bp × 2) using the Illumina HiSeq2500 platform. Approximately 15 Gb (SN, sufficient nitrogen) and 13 Gb (LN, limited nitrogen) of sequences were obtained. After removing low quality sequences below the Q20 threshold, short sequences less than 40 bp in length, and those with residual rRNA, 83.3 % SN and 67.3 % LN of the high-quality reads were successfully aligned against the maize B73 reference genome (V3) using the splice junction mapping algorithm in Tophat2 [49] (Table 1).

**Table 1:** Overview of two total RNA-seq datasets

Samples	Raw bases	Cleaned bases	Mean length	Mapped (%)
SN	15,179,396,400	14,534,219,565	97.02	83.3
LN	13,175,171,000	12,408,379,110	96.72	67.3

*SN* B73 under sufficient nitrogen condition, *LN* B73 under limited nitrogen condition

**Fig. 2:**
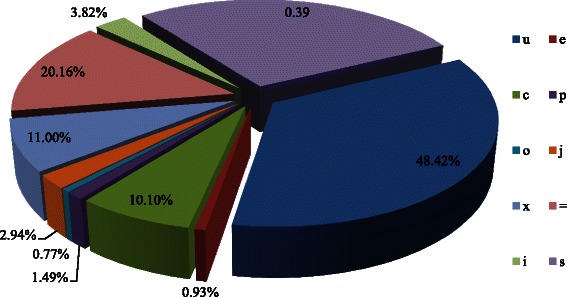
Annotation classification of assembled transcripts based on reference gene set. The percentage was calculated based on class codes generated by Cuffcompare against B73 reference gene set ( Ensembl v26). Among of class codes, “=”: locus completely matched with intron chain; “c”: locus contained in reference gene; “j”: locus is potentially a novel isoform and at least 1 splice junction is shared with a reference transcript; “e”: a possible pre-mRNA fragment; “i”: a transfrag falling within an intron region; “o”: generic overlap with reference; “p”: possible polymerase run-on fragment; “x”: exonic overlap with opposite strand of the reference; “s”: intronic overlap with opposite strand of the reference likely due to mapping error

To identify unannotated RNA transcripts, Cufflinks produced a merged data set of all nitrogen-treated RNA-seq data sets using the RABT (reference annotation-based transcript) assembly algorithm. As a result, 68,541 loci with 72,169 transcripts from B73 were generated. Comparison with the known B73 reference gene set (Plant Ensembl database, v26) by the Cuffcompare program produced a non-redundant combined set of 66,342 loci with 72,169 transcripts for further analysis. Meanwhile, the comparison results showed that 20.16 % (14,547) of transcripts were completely matched to annotated coding genes, while 34,945 (48.42 %) transcripts were mapped to unknown intergenic regions and 3.82 % (2755) transcripts were located entirely within a reference intron (Fig. 2). In total, 2119 (2.94 %) transcripts were presumed to be novel isoforms. The results indicated that the majority of the transcripts were from the intergenic or intronic regions, and the total RNA-seq strategy is capable of recovering these RNAs.

### Computational identification and characterization of intergenic and intronic lncRNAs

**Fig. 3:**
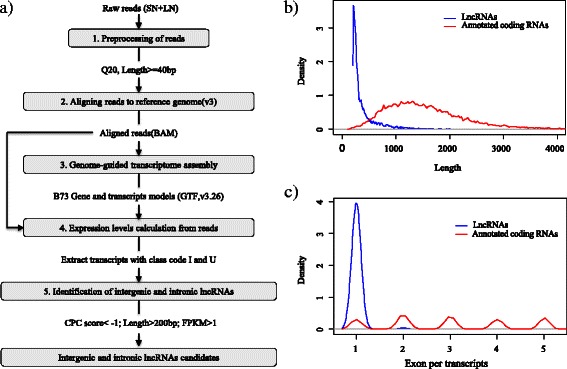
Flow chart of intergenic and intronic lncRNA identification and these length/exonic density. **a** Flow chart of intergenic/intronic lncRNAs and novel protein-coding gene identification analysis. RNA-seq data sets were assembled and merged into a transcriptome by Cufflinks. CPC score, protein domain and length were used to set inclusion and exclusion criteria for screening intergenic/intronic lncRNAs and putative protein-coding RNAs among the candidate unannotated transcripts. **b** Density plot of lengths for lncRNAs (blue) and coding RNAs (red). **c** Density plot of exons per transcript for lncRNAs (blue) and coding RNAs (red)

Potential lncRNAs and novel protein-coding mRNAs were identified based on their sequence, amino acid peptide lengths and protein-coding potential, using CPC against UniRef90 and Pfam protein databases (Fig. 3a). In this report, only intronic and intergenic transcripts (“I” and “U”, respectively) were selected for lncRNA prediction. Of the 37,700 transcripts with class code “I” and “U”, 16,516 were identified with non-coding transcripts after CPC analysis (CPC score < -1). Non-coding transcripts were aligned against Pfam protein databases for domain filtering. Finally, 7245 non-coding transcripts longer than 200 bp and with an FPKM > 1 were considered as LncRNAs and 5481 were potential novel protein-coding transcripts (CPC score > 1) (Additional file 3: Table S3 and Additional file 4: Table S4).

**Fig. 4:**
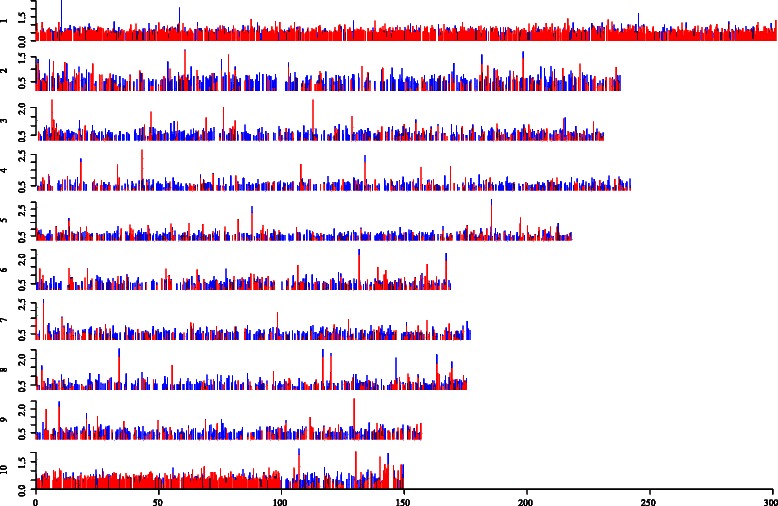
Chromosome distribution and expression value of intergenic/intronic lncRNAs under nitrogen treatments. The expression values of intergenic/intronic lncRNAs were identified in this study (y-axis) across the genome (x-axis). Blue represents the log_10_(FPKM + 1) value per intergenic/intronic lncRNAs with high nitrogen treatment; Red represents the log_10_(FPKM + 1) value per intergenic/intronic lncRNAs with low nitrogen treatment

Among the 7245 putative lncRNAs, most were intergenic transcripts (6,211 lncRNAs) (class code “U”), while 1034 were located fully within an intron of a protein-coding gene (class code “I”) (Additional file 3: Table S3). The average length of lncRNAs was shorter than that of coding RNAs and mostly had only one exon (Fig. 3b, c). LncRNAs from intergenic regions could also be named as long intervening non-coding RNAs (lincRNAs). According to their genome positions, these lncRNAs overlapped with parts of inter- and intragenic sequences and were widely distributed in maize genome (Fig. 4). A fasta-formatted file containing the identified lncRNAs file and a GTF-formatted annotated file are provided in Additional file 5: Table S5.

Moreover, we used Megablast [58] to identify sequence similarities with known plant ncRNAs (PNRD, plant non-coding RNA database [63]). As a result, 167 intergenic/intronic lncRNAs showing high homology to known lncRNAs from PNRD lncRNA databases were considered as known lncRNAs. In addition, four miRNA precursors, two tRNAs, one snRNAs and 16 snoRNAs were identified as known ncRNAs (Additional file 6: Table S6).

### Evolutionary conservation of intergenic/intronic lncRNAs

To evaluate the sequence conservation, we investigated the sequence similarity relationships of intergenic/intronic lncRNAs among three monocot genomes obtained from the plant ensembl genome database (http://plants.ensembl.org/) using Blast [58] alignment. Annotated coding RNAs (class code, “=”) sequences served as a control. Distributions of Blast scores were then visualized using SimiTri [64] (Fig. 4, Additional file 7: Table S7).

**Fig. 5:**
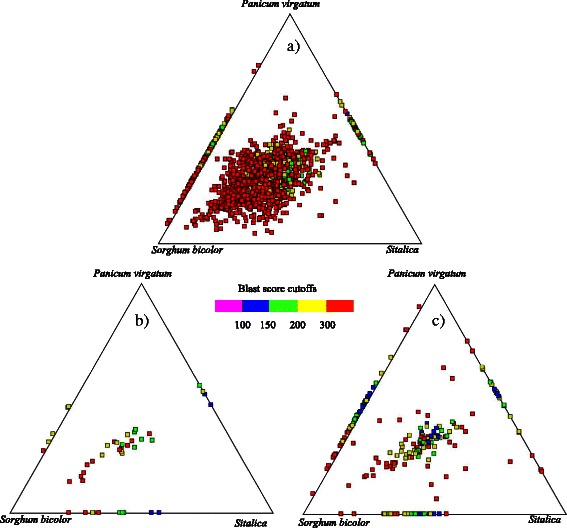
Similarity profiles of lncRNAs and annotated coding genes across related species. The nitrogen-responsive linRNA were searched separately against panicum virgatum,Sorghum bicolor and Sitalica genomes using Megablast (E-value ≤ 1e-20). The color was coded based on the highest BLAST score to each database: red ≥ 300; yellow ≥200; green ≥150; blue ≥100, and purple <100. **a** Annotated coding RNAs with class code “=”; **b** putative intronic lncRNAs; **c** putative intergenic lncRNAs

The extent of sequence similarity observed among intergenic and intronic lncRNAs was higher than that seen in randomly selected intergenic regions. However, both intergenic and intronic lncRNAs appeared to show less similarity among the different genomes compared with the annotated coding RNA set (Fig. 5a, b, c). A Mann-Whitney *U* test showed that sequence similarity of the intergenic/intronic lncRNAs was significantly different from the corresponding set (*p* = 2.2 × 10^−12^).

### Unique expression pattern of lncRNAs

**Fig. 6:**
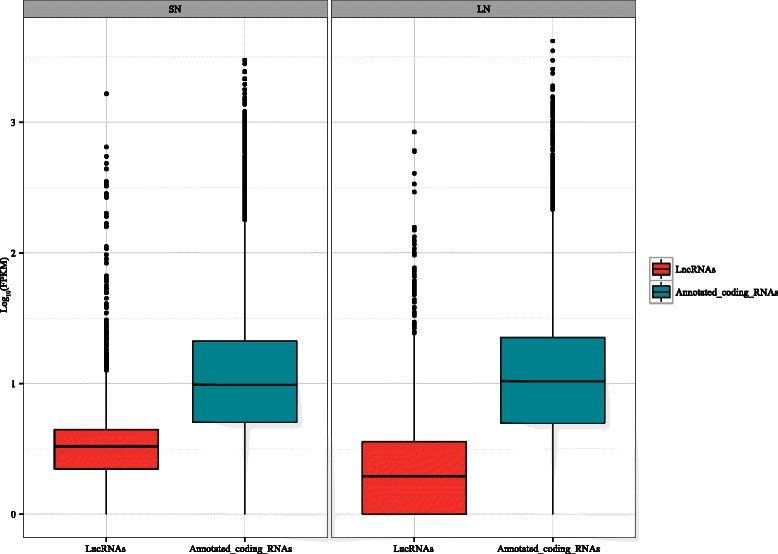
Comparison of expression pattern of intergenic/intronic lncRNAs and annotated coding RNAs. Expression values (Log_10_ (FPKM + 1 ))of intergenic/intronic lncRNAs cluster, novel coding RNAs cluster and annotated RNAs cluster were calculated separately based on SN and LN RNA-Seq data sets. A horizontal line represents the median of each sample

According to RNA types, expressed transcripts were classified into two major clusters: the lncRNAs group and the annotated coding RNAs group. The expression patterns of the two clusters were calculated separately. In detail, the difference between lncRNAs and coding RNAs was measured statistically using a two-tailed Mann-Whitney *U*-Test (Fig. 6, Additional file 8: Table S8).

The expression pattern of lncRNAs showed significant differences to that of the coding genes (*p* < 2.2e^-16^ in both SN and LN). The results suggested lncRNAs have their own unique expression patterns and their overall expression levels were significantly lower than those of coding RNAs.

### Nitrogen-responsive intergenic and intronic lncRNAs

**Fig. 7:**
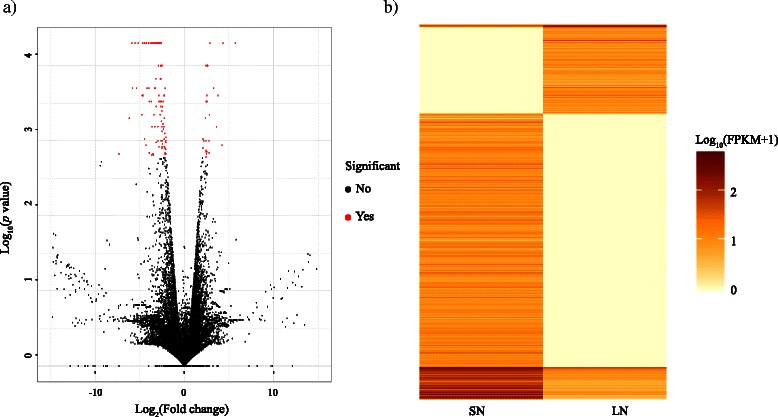
Volcano and heatmap plot of expressed transcripts between two nitrogen conditions. **a** Volcano plot of expressed information of isoforms. The red points indicate that both large-magnitude fold-changes (x-axis) as well as high statistical significance (-log_10_ of p-value, y-axis). **b** Heatmap plot of differential expressed isoforms with *p* < 0.01

To investigate the potential role of intergenic and intronic lncRNAs under nitrogen stress, we performed differentially expressed genes analysis between samples with different N treatments. Based on combined GTF annotation, all transcripts’ expression profiles were obtained by Cufflinks. Differentially expressed genes were analyzed by Cuffdiff and 2391 transcripts with q values < 0.05 were identified as differentially expressed. In detail, 637 of these differentially expressed transcripts were from intergenic and intronic lncRNAs (620 intergenic and 17 intronic RNAs). Under high nitrogen conditions, 426 intergenic/intronic lncRNAs were downregulated, including 417 intergenic and nine intronic RNAs, while 211 lncRNAs (203 lincRNAs and eight intronic lncRNAs) were upregulated (Fig. 7, Additional file 9: Table S9). The remaining 1307 were other differentially expressed transcripts. Meanwhile, we observed that 6608 lncRNAs were statistically insignificant under the two nitrogen treatments. Differentially expressed lncRNAs were further evaluated using co-expression analysis to infer their potential function.

### Co-expression network of nitrogen-responsive lncRNAs

Compared with specific coding genes and microRNAs, most lncRNAs’ functions, particularly in response to nitrogen resources, remain largely unknown. Many reports have suggested that co-expressed genes are usually members of the same pathway or protein complexes and are functionally related or controlled by the same transcriptional regulatory program [65–67]. Genes or proteins inside a co-expressed module can be co-regulated. Therefore, computational construction of coding and non-coding gene co-expression networks could be used to infer the potential biological functions of the lncRNAs [66].

**Fig. 8:**
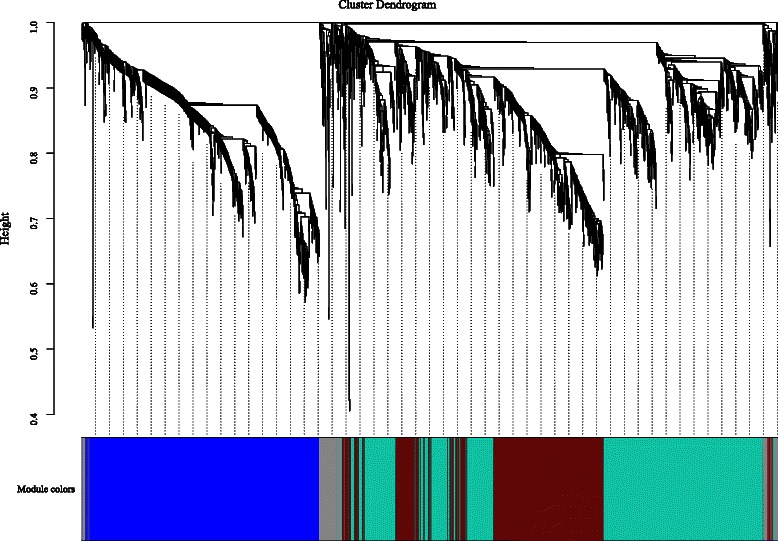
The co-expression network between nitrogen-responsive intergenic/intronic lncRNAs and coding genes. Clustering dendrogram of lncRNA and coding genes, with dissimilarity based on topological overlap, together with assigned module colors. Module colors are assigned according to module size: turquoise denotes the largest module, blue next, then brown, green, yellow, etc. The color grey is reserved for non-module genes

**Fig. 9:**
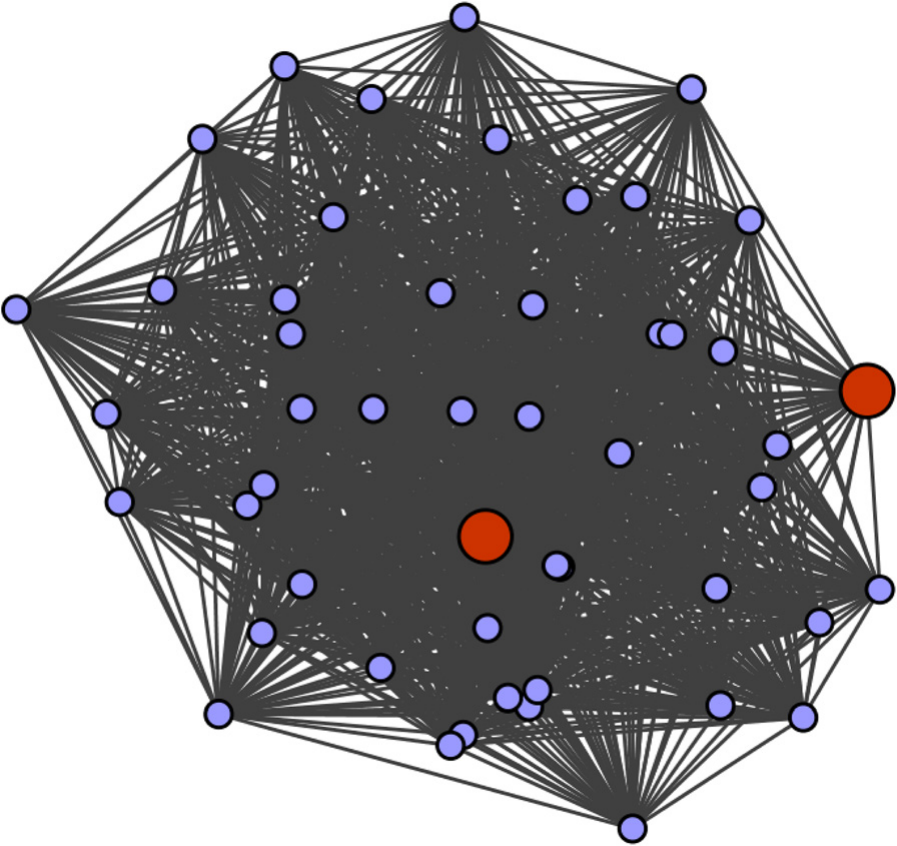
Graphical representation of coexpressed network M1 (weight threshold = 0.02). Top 50 nodes representing genes with high intramodular connectivities from M1 modules were extracted with weight threshold 0.02 and exported to an edge file and a node file for visualization by Cytoscape. Red represents lncRNA nodes; Blue represents other nodes

After similarity analysis of microarray probes with E values < 1e^-50^, 85 probes were identified and considered as differentially expressed intergenic/intronic lncRNAs (Additional file 10: Table S10). We used a well-developed computational algorithm, WGCNA, to construct the co-expression network for coding RNAs and lncRNAs. The distribution of association between coding genes and lncRNA genes was calculated (Pearson correlation, *R*^*2*^ ≥ 0.85). The significantly correlated genes were selected to construct the co-expression network. Excluding the reserved non-coexpressed gray module, we dissected three modules that included 33 lncRNAs that comprise various nodes in the network. Interestingly, we noted that most lncRNAs (32 of 33) were enriched in the blue module M1 (Fig. 8, Table 2); therefore, we focused on analyzing module M1. Module M1 comprises 32 N-responsive intergenic and intronic lncRNAs and 239 maize protein-coding genes (Megablast against maize annotated coding genes with e-value ≤ 1e^-50^) (Additional file 11: Table S11). Using intramodular connectivity calculations, two lncRNAs: TCONS_00068420 (A1ZM007479_at, *E* value = 6e^-96^) and TCONS_00020903 (A1ZM007576_at, *E* value = 2e^-121^) exhibited very high intramodular connectivity, which suggested that these lncRNAs play an important hub role in regulating the eigengenes of module M1 (Fig. 9). A full list of the genes in the blue modules is provided in Additional file 11: Table S11.

**Table 2:** The statistics of coding genes and lncRNAs in constructed co-expressed modules

Modules	M1 (blue)	M2 (turquoise)	M3 (brown)
Other probes	904	1053	665
LncRNAs probes	32	0	1
Total probes	936	1053	666

M1 ~ M3 represent the constructed co-expressed modules respectively

### Function term enrichment of M1 eigengenes

**Fig. 10:**
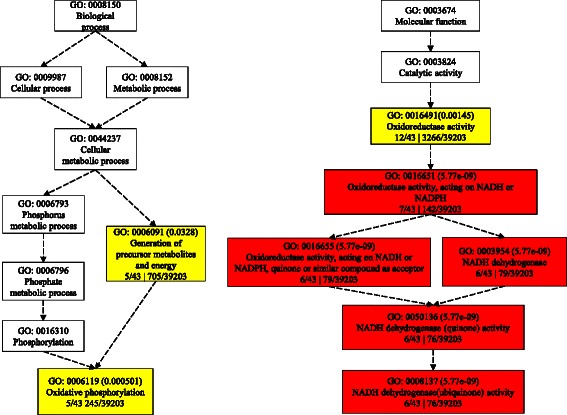
The network of enriched GO terms. The color represents the significant levels from yellow to red

The coding eigengenes of module M1 were further assigned and enriched to different GO terms using AgriGO [60] toolkits. We observed some significantly enriched genes in the biological process category of oxidative phosphorylation (GO: 0003954, corrected *p* value < 0.0005), generation of precursor metabolites and energy (GO: 0006091, *p* value < 0.033) and oxidation reduction (GO: 0055114, *p* < 0.054). In the molecular function category, NADH dehydrogenase activity (GO: 0003954, *p* < 5.80e^-09^) and oxidoreductase activity (GO: 0016491, *p* < 0.0033) were highly and significantly enriched (Fig. 10, Table 3). Besides, eigengenes were also assigned to GO terms such as phosphorylation (GO: 0016310), phosphate metabolic process (GO: 0006796), phosphorus metabolic process (GO: 0006793), nitrogen compound metabolic process (GO: 0006807), biosynthetic process (GO: 0009058) and cellular macromolecule metabolic process (GO: 0044260) (Additional file 12: Dataset S1).

**Table 3:** Top 15 annotated and enriched GO terms of M1 module eigengenes

GO term	Ontology	Description	*p*-value	FDR
GO:0003954	F	NADH dehydrogenase activity	4.00E-10	5.80E-09
GO:0016651	F	oxidoreductase activity, acting on NADH or NADPH	2.80E-10	5.80E-09
GO:0006119	P	oxidative phosphorylation	0.000008	0.0005
GO:0016491	F	oxidoreductase activity	0.00015	0.0014
GO:0006091	P	generation of precursor metabolites and energy	0.001	0.033
GO:0055114	P	oxidation reduction	0.0026	0.054
GO:0003824	F	catalytic activity	0.014	0.12
GO:0016310	P	phosphorylation	0.029	0.45
GO:0006796	P	phosphate metabolic process	0.043	0.45
GO:0006793	P	phosphorus metabolic process	0.043	0.45
GO:0006807	P	nitrogen compound metabolic process	0.5	1
GO:0006139	P	nucleobase, nucleoside, nucleotide and nucleic acid metabolic process	0.66	1
GO:0044260	P	cellular macromolecule metabolic process	0.96	1
GO:0044238	P	primary metabolic process	0.82	1
GO:0016740	F	transferase activity	0.61	1

### Validation and quantification of lncRNAs

**Fig. 11:**
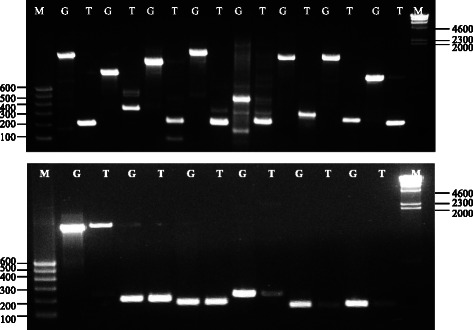
PCR test against Genomic DNA and cDNA. M: Marker; G: Genomics DNA; T: cDNA transcripts

**Fig. 12:**
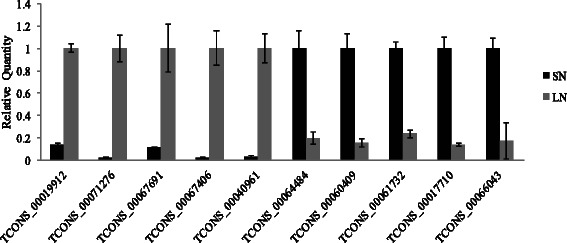
qPCR results of randomly selected N-responsive transcripts. X-axis represents selected 10 differential expressed transcripts under N stress. Here, ubiquitin was chosen as the reference gene. Relative expression value per selected transcripts between SN and LN samples was calculated (y-axis)

We selected 14 lncRNAs, eight with introns and six without introns, to conduct PCR validations using genomic DNA and cDNA. The results showed a high degree of consistency for the product sizes between assembled lncRNAs and the actually amplified product, both at genomic and transcriptome levels (Fig. 11, Additional file 1: Table S1). The results excluded the possibility of mis-assembly of sequences and alternative splicing. To ensure the accuracy and reliability of the RNA-seq results, a set of independent biological replicates of the nitrogen treatments were subjected to qPCR to confirm the expression changes. Ten transcripts were randomly selected for qPCR (Fig. 12, Additional file 2: Table S2). The results showed good consistency between the qPCR results and the high-throughput sequencing results.

## Discussion

Compared with traditional microarrays, RNA-seq is superior for the detection of novel lncRNAs because of its greater sensitivity, high throughput and no need for prior sequence information [68, 69]. In mRNA sequencing, the mRNAs are captured based on the presence of a poly (A) tail. Thus, lncRNAs with polyA tails can be captured. However, Cheng et al. [70] suggested that 40 % of lncRNA transcripts were not polyadenylated. To obtain a global view of lncRNAs, we constructed and sequenced an entire RNA library. Only ribosomal RNAs were removed. The small RNAs, mRNAs, and all forms of lncRNAs were retained. As a result, we detected more lncRNAs than any other reported mRNA sequencing projects.

Although recent studies of lncRNAs suggested that individual lncRNAs might play important and diverse biological roles, only a few plant lncRNAs have been confirmed to regulate abiotic stress. In this study, we surveyed the intergenic/intronic lncRNAs in B73 leaves at the V7 stage under conditions of N-deficiency and N-sufficiency. Integrated with mRNA expression profiles and physiological evaluations, 7245 lncRNAs and 637 nitrogen responsive lncRNAs were found. Moreover, a number of lncRNAs were identified for the first time as specifically expressed under different N conditions. The highly specific temporal and spatial expression pattern is similar to previous observations in other plant species [44].

The functions of most lncRNAs remain largely unknown, therefore, we constructed a gene co-expression network of nitrogen-responsive lncRNAs and coding genes and identified three modules using public microarray data sets under different nitrogen treatments. Interestingly, co-expressed lncRNAs were mainly clustered in module M1. Thus, lncRNAs may play a key role in gene expression regulation of module M1. Further functional enrichment results suggested associations with oxidation and reduction, generation of precursor metabolites and energy processes. This result indicated that N treatment during maize fertilization could have a profound influence on the energy and substrate metabolism in leaves, which is highly consistent with the expectation that the category of nitrogen compound metabolic process (GO: 0006807) could be found in the N treatment experiment. However, the most significant process was NADPH/NADH dehydrogenation (GO: 0003954, GO: 0050136, GO: 0008137, GO: 0016655 GO: 0016651). NADH and NADPH are the reduced forms of NAD+ and NADP+, respectively. The assimilation of nitrogen is associated with high NADH/NADPH consumption [2]. N nutrient absorption improves the photosynthesis system which is one of the biggest resources of NADPH production in plants [71]. NADPH also acts as an electron donor in carbon dioxide fixation in the Calvin cycle (light-independent reactions) [72] and lipid biosynthesis [73]. Phosphorylation (GO: 0016310) was also found, which is a vital procedure in carbon fixation, and in lipids and starch assembly. Previous studies showed that plant growth and biomass production are largely connected to the activity of primary metabolism in the source leaf, including carbon fixation in photosynthesis, large amounts of nitrogen for amino acids and proteins, phosphorus for the synthesis of RNA and the realization of energy metabolism. Environmental stress is usually accompanied by a rebalancing of the cellular C-N-P homeostasis [74] in plants. The annotated results in this study offered new insights into the potential roles of lncRNAs in C-N-P rebalancing in response to nitrogen.

One limitation is that only 85 lncRNAs were represented by microarray probes. With the rapid accumulation of total RNA-seq data, we hope that a set of combined expression data containing all lncRNAs will soon be available, which would allow the construction of a more comprehensive co-expression network in the future.

Our current understanding of lncRNA regulation in response to nitrogen is still in its infancy. Several approaches can be used to determine their biological functions, including lncRNA silencing and structure disruption. The present study has laid a foundation for future research in this direction.

## Conclusion

Genome-wide identification, characterization, differential expression and co-expression network analysis of intergenic/intronic lncRNA in maize leaves provided a global overview of transcriptional responses of lncRNAs to N stress. Co-expression analyses suggested that the expression of N responsive lncRNAs is highly enriched in a co-expressed module that is related to energy metabolic pathways. Future efforts will be devoted to understanding the interaction of these nitrogen-responsive lncRNAs, especially those with hub lncRNA functions in the network module. Experimental approaches such as overexpression, RNA interference and promoter analysis have been demonstrated as useful strategies to characterize their functions, which would provide valuable information for nitrogen-use efficiency improvement in maize.

### Availability of supporting data

The data set supporting the results of this article is available in the NCBI SRA database as SRP059655.

### Ethics approval and consent to participate

Not applicable.

### Consent for publication

Not applicable.

## Additional files

Additional file 1: Table S1. The 7524 identified intergenic and intronic lncRNAs. (XLSX 555 kb) Additional file 2: Table S2. The 5481 identified novel coding RNAs. (XLSX 346 kb) Additional file 3: Table S3. Annotation results based on known ncRNA database. (XLSX 26 kb) Additional file 4: Table S4. Blast similarity results of lncRNAs and annotated coding genes across related species. (XLSX 489 kb) Additional file 5: Table S5. Expression information of lncRNAs and annotated coding RNAs. (XLSX 2992 kb) Additional file 6: Table S6. Differentially expressed transcripts between two N conditions. (XLSX 76 kb) Additional file 7: Table S7. The 85 corresponding probes of N-responsive lncRNAs. (XLSX 17 kb) Additional file 8: Table S8. 239 coding genes among eigengenes of the M1 module. (XLSX 34 kb) Additional file 9: Table S9. Enriched GO terms of eigengenes of the M1 module. (XLSX 11 kb) Additional file 10: Table S10. Primers from 14 lncRNAs for PCR testing. (XLSX 15 kb) Additional file 11: Table S11. Primers from randomly selected 10 differentially expressed RNAs for qPCR testing. (XLSX 15 kb) Additional file 12: Dataset S1. The FASTA-formatted sequences file of identified lncRNAs and the GTF-formatted annotation file of identified lncRNAs. (ZIP 1290 kb)

## Abbreviations

ATP: adenosine triphosphate
FDR: false discovery rate
FPKM: fragments per kilobase per million mapped reads
GO: Gene Ontology
lincRNAs: long intervening non-coding RNAs
LN: limited nitrogen
lncRNA: long non-coding RNA
N: nitrogen
rRNA: ribosomal RNA
SN: sufficient nitrogen
